# Discovering the fibroblastic reticular cell in the immune tumor microenvironment in lymphoma

**DOI:** 10.1172/JCI171310

**Published:** 2023-07-03

**Authors:** Adam Yuh Lin, Leo I. Gordon

**Affiliations:** 1 Division of Hematology and Oncology, Department of Medicine, Feinberg School of Medicine, Northwestern University, Chicago, Illinois, USA.; 2 Robert H. Lurie Comprehensive Cancer Center of Northwestern University, Chicago, Illinois, USA.

## Abstract

The study of the cellular and molecular microenvironment in B cell lymphoma, especially diffuse large B cell lymphoma (DLBCL), has led to prognostic and therapeutic algorithms that may improve patient outcomes. Emerging gene signature panels provide a granular understanding of DLBCL based on the immune tumor microenvironment (iTME). In addition, some gene signatures identify lymphomas that are more responsive to immune-based treatment, indicating that the iTME has a biological signature that could affect outcomes when targeted. In this issue of the *JCI*, Apollonio et al. report on fibroblastic reticular cells (FRCs) as potential targets in aggressive lymphoma. FRCs interacted with lymphoma cells and induced a state of chronic inflammation that suppressed immune function by impeding optimal T cell migration and inhibiting CD8^+^ T cell lytic function. These findings suggest that manipulating the iTME by directly targeting FRCs may enhance responses to immunotherapy in DLBCL.

## FRCs and resistance mechanisms of immunotherapy

Fibroblastic reticular cells (FRCs) are a specialized subset of stromal cells found in lymphoid organs. They play an essential role in immune response regulation and immune cell trafficking, retention, and activation by intimate crosstalk with various immune cells, including T cells, B cells, and dendritic cells (1). First identified as part of a host response to viral infections, they have since been found to have critical immunoregulatory properties. FRCs appear to augment CD8^+^ T cell differentiation into tissue-resident memory CD8^+^ cells by epigenetic remodeling (2). Thus, we now appreciate the role of the FRC in immune homeostasis. These findings have led to the investigation of FRCs as part of the host tumor microenvironment (TME) and therefore to studies in cancer research. Specifically, the observation that remodeling of FRCs could protect CD8^+^ T cells from exhaustion has sparked interest in studying the FRC in settings where T cell exhaustion may be a central part of the host response to cancer, such as in lymphoma. In lymphoma, the FRCs also serve as cancer-associated fibroblasts (CAFs). The crucial role of CAFs in the TME is well established. CAFs are responsible for matrix remodeling, immune crosstalk, metabolic effects, and soluble secreted factors to modulate cancer invasion, immune cell–endothelial cell interactions, and tumor growth (3). With the recent emergence of cellular therapy in cancer and especially in lymphoma, the role of T cell exhaustion as a mitigating factor has become a focus of research.

**Figure 1:**
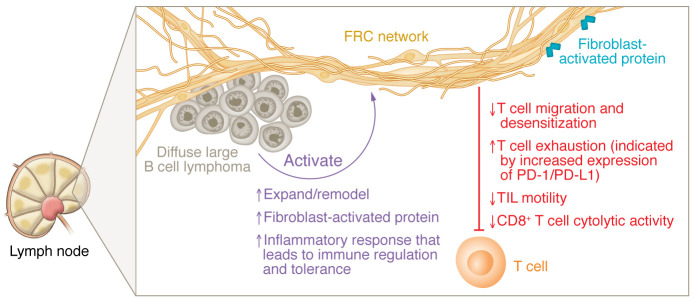
FRCs modulate the iTME. In the setting of DLBCL, lymphoma cells activate FRCs to undergo expansion and modulation, expression of FAPs, and enhancement of inflammatory pathways, which regulate immune cells in a chronic inflammatory setting. Apollonio et al. (4) propose these changes lead to modulations of the iTME by slowing T cell migration to the tumor, increasing exhaustion markers, reducing TIL motility, and reducing CD8^+^ T cell cytotoxicity.

FRCs, therefore, are a potential target in aggressive lymphoma. In this issue of the *JCI*, Apollonio and authors used models including lymph nodes from healthy patients, lymph nodes from patients with diffuse large B cell lymphoma (DLBCL) (4), and a murine model with BCL6 expression that was first developed by the Dalla-Favera group (5). Apollonio and authors found that the DLBCL-related FRCs had an altered transcription state that resembled activated cells of the TME immune system. A similar transcription pattern follows viral infection, with increased expression of genes involved in proliferation, metabolism, type I and II interferon signaling, and antigen presentation. In addition, they found that lymphoma-exposed FRCs inhibited CD8^+^ TILs, which was reversible with the bispecific antibody glofitamab. Notably, the role of so-called “bystander” lymphocytes (tumor-infiltrating lymphocytes [TILs]) has been explored as a strategy in cancer therapy (6). Bystander TILs are activated in a TCR-independent manner and do not recognize infection-related or cancer-specific antigens. Adding checkpoint inhibitors (CPIs), chimeric antigen receptor (CAR) T cells, and bispecific antibodies may engage TILs to increase responses (7). The findings from Apollonio et al. suggest that the interaction between FRCs and lymphoma cells induces a state of chronic inflammation that suppresses immune function by impeding cell migration and inhibiting CD8^+^ T cell lytic processes (4) (Figure 1).

## Clinical implications of immune TME in lymphoma

TME fibroblasts are better studied in solid tumors (8), but important insights into cellular therapy may be relevant in lymphoma. While treatment of non-Hodgkin’s lymphoma with immune-based approaches such as CPIs has yielded disappointing outcomes (9, 10), attempts to understand better the role of the exhausted CD8^+^ T cell population in mediating responses offer hope for improvement. Investigation of the TME in lymphoma has provided a basis for understanding and predicting responses to CPI. The TME in lymphoma is thought to show either an inflamed or noninflamed phenotype (11). The inflamed TME, for example, in classical Hodgkin’s lymphoma (cHL) and some primary mediastinal B cell lymphomas (PMBCLs) is characterized by immune cell infiltration and, at the molecular level, by copy gains of chromosome 9p24.1, which encodes the programmed cell death ligand 1 (PD-L1) locus and is selectively amplified (12). Recent data show that CPI therapy is a highly effective treatment for patients with cHL (13). It may even be the preferred choice for cHL patients, whether as a first or second line of therapy (14). In contrast, most cases of non-Hodgkin’s lymphoma can be considered noninflamed, and responses in DLBCL treated with CPI are less successful. Apollonio et al. (4) proposed DLBCL as having an inflamed phenotype, yet it is unclear why the TME in DLBCL does not respond better to CPI, as observed in cHL or PMBCL. Specifically missing in DLBCL are the 9p24.1 chromosome gains that are seen in cHL. Manipulation of the TME by targeting the FRC, as described by Apollonio et al. (4), may provide one way of enhancing responses to CPI in lymphoma.

## Cellular therapy and bispecific antibodies

CAR T cell therapy has opened avenues in lymphoma therapy (15), especially for patients with DLBCL who previously had limited options. Yet only 35% to 40% of patients achieve long-term complete remissions (16–18). It is critical to explore the causes of treatment response and failure. Current research has focused, among other areas, on the immune tumor microenvironment (iTME). Attributes of the iTME that predict CAR T cell efficacy include the loss of target antigen, T cell exhaustion (19–21), a rapid change in immune TME after treatment, a pretreatment TME rich in T cell–related cytokines, and a high density of PD-1^+^LAG-3^+/–^ T cells (21). Low numbers of preinfusion Tregs in the TME correlate with increased neurological toxicity but better responses. Tregs (FOXP3^+^CD25^hi^), therefore, play a major role in determining response to CAR T therapy and may serve as biomarkers. T cell exhaustion parameters are inversely correlated with circulating CAR T numbers (21). Further, markers of T cell activity could be predicted by a TME that was enriched for CCL5 and CCL22 as well as γ chain receptor cytokines such as IL-15, IL-7, IL-21, and interferon-regulated molecules (21). Combination of a tumor-targeted fibroblast-activated protein (FAP) bispecific antibody with the CD3- and CD20-targeted bispecific antibody glofitamab can target FRCs and may have translational potential (7). Glofitamab as a single agent has shown great promise in B cell lymphoma. Appropriate timing and combinations of these antibodies and biomarkers may overcome some inherent immunologic barriers to success (7).

## Summary and the way forward

These studies and similar investigations of the TME, including the understudied FRC, should have an impact and enhance our understanding of some of the immunologic and cellular therapies that are entering the clinical arena for B cell lymphomas. Manipulation of the TME may well be a part of future therapy in lymphoma and, more broadly, in cancer therapy over the next decade. Further studies using in vivo models to evaluate response and toxicity will be critical to understanding the clinical implications for targeting FRCs. Targeted FRCs might enhance other modalities such as armored CAR T cells, dual-targeting CAR T cells (22), IL-7 antibody combination with CAR T cells (23), EZH2 inhibitors with bispecific antibodies (24), and allogeneic CAR T cells (25). Manipulating the Treg population and using Tregs as biomarkers appear central to improving outcomes after CAR T therapy in lymphoma. Therefore, further studies investigating the FRC and other components of the iTME in B cell lymphomas are warranted.

## Version 1 07/03/2023

Electronic publication
